# Clinical presentation and outcome of tuberculosis in chronic kidney disease stage 4 & 5 from a high TB burden country

**DOI:** 10.1371/journal.pone.0320907

**Published:** 2025-04-02

**Authors:** Sunil Kumar Dodani, Zaheer Udin Babar, Khadija Gul Mohammad, Saima Ali, Maryam Mushtaq, Salma Batool, Ali Nadeem, Asma Nasim

**Affiliations:** 1 Department of Infectious Diseases, Sindh Institute of Urology and Transplantation, Karachi, Pakistan; 2 Department of Nephrology, Sindh Institute of Urology and Transplantation, Karachi, Pakistan; 3 TB Center, Provincial TB control Program, Sindh Institute of Urology and Transplantation, Karachi, Pakistan; 4 Department of Molecular Biology, Sindh Institute of Urology and Transplantation, Karachi, Pakistan; 5 Department of Microbiology, Sindh Institute of Urology and Transplantation, Karachi, Pakistan; International Centre for Diarrhoeal Disease Research, BANGLADESH

## Abstract

**Background:**

Diagnosis and management of Tuberculosis (TB) in chronic kidney disease (CKD) is challenging. Our aim is to study clinical presentation and outcome in patients with stage 4 & 5 CKD from a high TB burden country.

**Methods:**

All patients registered in Provincial TB Centre in tertiary care hospital in Pakistan from May 2016 to June 2020 were included. TB cases tested rifampicin resistant (RR) in GeneXpert were excluded from the study. Patients with CKD stage 4 & 5 were studied for demographics, TB history, clinical feature, diagnoses, treatment success and mortality. CKD stage 4 & 5 were compared with other patients registered at the TB treatment center.

**Results:**

Out of 828, 259 (31%) had CKD stage 4 & 5. Out of 259, 156 (60%) had extra-pulmonary TB (EPTB). Microbiological diagnosis done in 118 (45.51%), 25% in EPTB and 72.9% in pulmonary TB (PTB). TB culture was positive in 46 (17.8%), Isoniazid resistance 21.7%. Treatment success was 80.7%. PTB was significantly associated with mortality (p = 0.031). In CKD stage 4 & 5 treatment success was significantly lower with high mortality (p = 0.033).

**Conclusion:**

In CKD stage 4 & 5, EPTB is the most common presentation. Microbiological diagnosis could be achieved in one fourth of EPTB. There is high INH resistance. The treatment success is low with high mortality and PTB is a significant risk factor for mortality.

## Background

The incidence of non-communicable diseases like diabetes mellitus, hypertension and chronic kidney disease(CKD) is increasing both in low and high income countries [1] The prevalence of CKD in South Asia ranges from 10.6% in Nepal to 23.3% in Pakistan [2]. Patients with CKD and end stage renal disease (ESRD) have 3–25 fold increase risk of developing tuberculosis (TB) [3]. CKD and ESRD patients have impaired immune response due to complex mechanisms like uremia, malnutrition and disruption of cell mediated immunity which is primarily responsible for identification and killing of intracellular pathogens like *mycobacterium tuberculosis* [4]. A retrospective study by Shu et al found that risk of TB is higher in patients with stage 3 to 5 CKD and this risk is even higher among dialysis dependent patients [5]. Due to their partial immunocompromised state, clinical manifestations are often atypical, leading to inaccurate or delayed diagnoses. Moreover, treating TB in this population poses significant challenges, including: difficulties in administrating anti-TB medications due to need for adjustment in renal impairment, drug- drug interactions, and drug side effects [6]. As a consequence, the overall mortality rate for TB among CKD patients is alarmingly high, reaching up to 35% according to Xiao et al. [1].

Tuberculosis in CKD patients has significant public health implications in low- to middle-income countries like Pakistan, where CKD prevalence is rising and TB burden remains high. To inform evidence-based guidelines for diagnosis and treatment, a clear understanding of the relationship between these two conditions is crucial. However, data from Pakistan are scarce. A single-center study by Qureshi et al. addressed this knowledge gap, revealing that among 3000 dialysis-dependent patients, 2% had TB and 79% presented with extrapulmonary involvement. These findings underscore the need for further research and context-specific guidelines to effectively manage TB in CKD patients in Pakistan.

Our aim is to study clinical presentation and treatment outcome of TB in patients with moderate to severe renal impairment (stage 4 and 5 CKD).

## Methods

A retrospective cohort study was done at a tertiary care hospital from May 2016 till June 2020. Our hospital is one of the largest urology and dialysis center where more than 1000 dialysis sessions performed in a day. The hospital is also a specialized provincial TB treatment center working in collaboration with government TB control program. We provide first line drug sensitive TB treatment at our center. All patients diagnosed as TB and got registered in TB control program of the hospital were included. Patients who were diagnosed as ‘rifampicin resistance detected’ on Gene Xpert MTB/RIF (GXP) test were referred to Multi drug resistant (MDR) TB center and were excluded from the study. TB diagnosis was made by primary physician along with Infectious disease specialist. The diagnosis was made on clinical characteristics followed by relevant investigations as described in the definitions below. TB was defined as pulmonary tuberculosis (PTB), extra pulmonary TB (EPTB) and disseminated TB (DTB) (refer the definitions).

Detailed characteristics of included patients were noted such as, demographics, previous history and family history of TB and clinical features, microbiological data including Ziehl-Neelson (ZN) stain, GXP and TB culture with sensitivity, imaging results like chest X ray, computerized tomographic scan, magnetic resonance imaging and histopathological findings of the tissues biopsied.

The treatment protocols were 2 months intensive phase: Isoniazid 5mg/kg OD, Rifampicin 10 mg/kg OD, Ethambutol (ETH) 15-20mg/kg OD (if creatinine clearance < 30 then alternate days/ after hemodialysis), Pyrazinamide (PZA) 20-25mg/kg (if creatinine clearance < 30 then alternate days/after hemodialysis) followed by 4 to 10 month continuation phase (depending upon site of involvement): Isoniazid 5mg/kg OD, Rifampicin 10 mg/kg OD. INH resistant TB was treated according to WHO guidelines [7].

Any side effects were noted. Primary outcome was treatment success (as defined below)and all-cause mortality.

Patients with CKD stage 4 and 5 (refer the definition) were compared with all other registered patients included among them were patients with CKD stage 1-3. Following parameters were compared age, gender, site of TB disease, past history of TB, family history of TB, TB cultures with drug resistance and outcome.

All the patient’s data was anonymized by allotting a unique number to each file. Before conducting the file review, ethical clearance was obtained from ethical review committee of the hospital. The informed consent was waived by the committee. All data was accessed on 10^th^ June 2023.

### Operational definitions

**Pulmonary tuberculosis(PTB):** It is defined as patients having clinical features suggestive of PTB and positive sputum ZN or GXP assay or radiological findings suggestive of PTB in case of negative microscopy (defined as smear negative PTB).

**Extra pulmonary TB (EPTB)** diagnosis was made as per organ or system affected such as TB lymphadenitis, central nervous system TB, bones and joints TB, spinal (Pott’s disease), abdominal or genitourinary tract (GUTB) and pleural TB. Criteria for active EPTB diagnosis was made on the basis of positive ZN stain or GXP assay of any tissue or organ fluid such as ascitic fluid or pleural fluid or histopathological evidence of presence of granulomatous inflammation with caseous necrosis depending on suspected site involvement. Pleural, peritoneal and meningeal TB were also diagnosed on basis of clinical features and fluid analysis if ZN stain or GXP assay were negative.

**Disseminated TB (DTB)**: Patients having evidence of two or more than two (with or without PTB) system involvement on the basis of clinical, microbiological or radiological findings suggestive of TB.

**Clinical diagnosis:** TB diagnosed on clinical grounds with no microbiological evidence or anti-tuberculous treatment started by primary physicians on the grounds of pyrexia of unknown origin.

**Bacteriologically confirmed** **TB**: Acid fast bacilli smear positive and/or GXP positive on any sample and/or TB culture positive.

**Drug induced liver injury** will be diagnosed as bilirubin > 1.5mg/dl, SGPT > 3 times if symptomatic or > 5 times of upper limit of normal (ULN) level [8].

**Chronic kidney disease (CKD) Stage 4 and 5**: Patients diagnosed as CKD stage 4 or 5 by using CKD-EPI (Chronic Kidney Disease Epidemiology Collaboration) equation [9].

**Treatment success** was defined as patients completed the treatment with resolution of symptoms and had gained weight.

### Statistical analysis

SPSS version 20 was used to analyze the data. Normally distributed continuous variables were reported as mean ±  SD and non-normally distributed were reported as median with interquartile range. To compare the mean difference between groups, for continuous variables two sample t test was used whereas chi square, independent test or fisher exact test were used to determine proportion difference between groups. A cut off value of < 0.05 was determined as statistically significant difference.

## Results

Out of a total of 828 patients registered in TB center during the study period, 259 had CKD stage 4 & 5 disease, 102 had CKD 1-3 and 467 had normal renal functions.

Table 1 shows demographics, clinical features, diagnostic characteristics and outcome of the patients with CKD 4 & 5. The mean age was 34.68 ± 16.7 and 38.6% were female. A total of 156 (60%) had EPTB, the most common was TB lymphadenitis (n = 38; 14.7%). Sixteen patients (6.2%) had relapsed TB.

**Table 1:** Demographics, clinical features, diagnosis, drug resistance and outcome of patients with CKD stage 4 & 5. N = 259.

Characteristics	N (%)
**Age** (mean ± SD) years	34.68 ± 16.07
<18	43 (16.6)
19-34	93 (35.9)
35-60	107 (41.3)
>60	16 (6.2)
**Female**	100 (38.6)
**Past history of TB**	16 (6.2)
**Family history of TB**	17 (6.6)
**Clinical characteristics**	
Fever	139 (53.7)
Weight loss	138 (53.3)
**Site of disease**	
Pulmonary	96 (37.1)
Disseminated (pulmonary plus one other site)	7 (2.7)
Extra pulmonary	156 (60.2)
Lymphadenitis	37(14)
Genitourinary	33 (12.7)
Central nervous system	5 (1.9)
Vertebral osteomyelitis	18 (6.9)
Pleural	28 (10.8)
Abdominal	16 (6.)
Bone	1 (0.3)
Eye	2(0.7)
PUO	7 (2.7)
Others	9(3.4)
**Diagnosis**	
**Microbiological based**	118 (45.51)
Ziehl-Neelson stain	64 (24.7)
X-pert MTB/RIF assay	105 (40.5)
TB culture positive	46 (17.8)
**Histopathological based**	77 (29.7)
**Radiological based**	64 (24.7)
**Adverse side effects**	22 (8.5)
Drug induced liver injury	19 (7.3)
**Outcome** Treatment successfully completed	209 (80.69)
Lost to follow up	12 (4.6)
Mortality during treatment	38 (14.67)

Microbiological based diagnosis could be done in 118 (45.51%) patients. TB culture was positive in 46 (17.8%) samples out of which 35 (76%) were pulmonary specimens. Out of 46 positive cultures, 16 (34.78%) were resistant strains, 12 (26%) were mono and 4 (8.6%) were poly-drug resistant. INH resistance was 21.7%.

Treatment success was achieved in 209 (80.7%) patients. Overall 22 (8.5% patients experienced ATT induced side effects, out of whom 19 (86%) developed drug induced liver injury and required discontinuation or modification of regimen.

The mortality rate was 14.7%.

Table 2 shows comparison between CKD stage 4 & 5 and all other patients. In CKD stage 4 & 5 patients the treatment success was significantly lower with high mortality (p = 0.033).

**Table 2:** Comparison of TB in CKD stage 4 & 5 and other patients including CKD 1-3.

Characteristics	CKD stage 4 & 5 (n = 259) N (%)	Other patients including CKD stages 1-3 (n = 569) N (%)	*p*-value
**Demographics**			
Age years			
<18	43(16.6)	86 (15)	0.584
19–34	93 (35.9)	198 (34.9)	0.757
35–60	107 (41.3)	228(40)	0.736
>60	16 (6.2)	57(10)	0.071
**Gender**			
Male	159 (61.4)	319 (56)	0.150
Female	100(38.6)	250 (43.9)	
**Past history of TB**	16(6.2)	39(6.9)	0.717
**Family history of TB**	17 (6.6)	67 (11.8)	0.021
**Site of disease**			
Pulmonary	96 (37)	179 (31.5)	0.112
Disseminated	7(2.7)	25 (4.4)	0.242
Extra pulmonary	156 (60.2)	363 (63.8)	0.325
**TB culture positive**	46(17.8)	116(20.4)	0.377
**Drug resistant TB**	16/46(34.7%)	34/116 (29.3)	0.497
**Outcome**			
Treatment success	209 (80.7)	496(87.2)	0.033
Mortality	38(14.7)	50 (8.8)	
Lost to follow	12 (4.6)	23 (4)	

We compared survivors and non- survivors among CKD stage 4 & 5 patients (Table 3).

**Table 3:** Comparison between non-survivors and survivors among patients with TB and CKD Stage 4 & 5.

Characteristics	Non-survivors (n = 38) %	Survivors (n = 221) %	*p* = value
**Demographics**			
Age years			
<18	7(18.4)	36 (16.3)	0.744
19–34	13 (34.2)	80 (36.2)	0.813
35–60	13 (34.2)	94 (42.5)	0.336
>60	5 (13.2)	11 (5)	0.067^ŧ^
**Gender**			
Male	20 (52.6)	139 (62.9)	0.230
Female	18 (47.4)	82 (37.1)	
**Past history of TB**	1 (2.6)	15 (6.8)	0.480^ŧ^
**Family history of TB**	1 (2.6)	16 (2.7)	0.481^ŧ^
**Site of disease**			
Pulmonary	20 (52.6)	76 (34.4)	0.031
Disseminated	2 (5.3)	5 (2.3)	0.274^ŧ^
Extra pulmonary	16 (14.2)	140 (63.31)	0.013
**Drug resistant**			
Isoniazid	2 (5.3)	8 (3.6)	0.644
Rifampicin	0	1 (0.5)	1.00
Ethambutol	2 (5.3)	3 (1.4)	0.157^ŧ^
Pyrazinamide	2 (5.3)	2 (0.9)	0.104^ŧ^
Mono resistant	2 (5.3)	10 (4.5)	0.691^ŧ^
Poly resistant	2 (5.3)	2 (0.9)	0.104^ŧ^

^ŧ^ = Fisher’ exact test.

Pulmonary TB was significantly associated with mortality (52.6 vs 34.4 *p* = 0.031).

**Fig 1:**
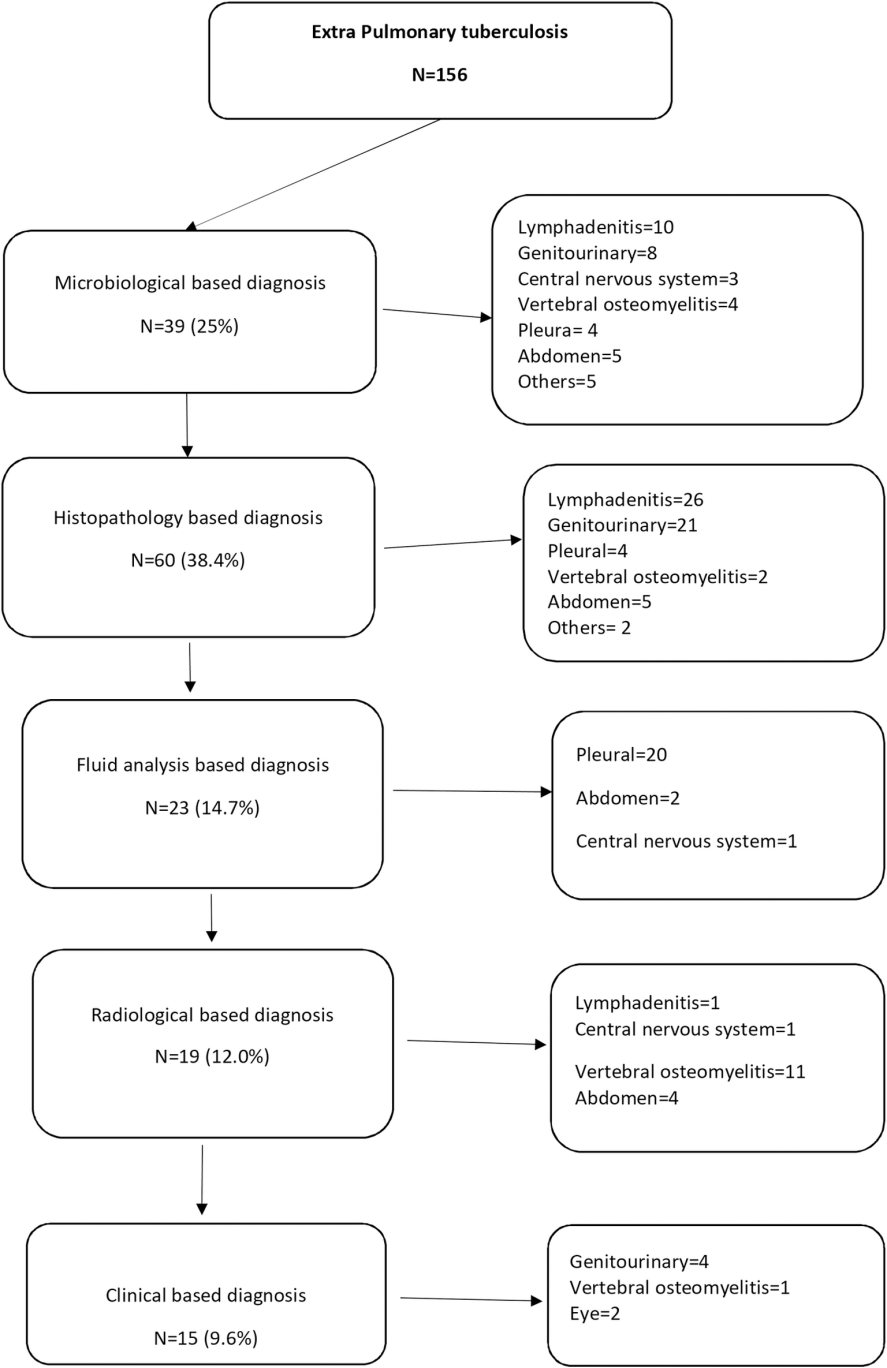
EPTB diagnosis in CKD stage 4 & 5.

The detailed methods of diagnoses in EPTB is shown in Fig 1. The most common way of diagnosis in EPTB was histopathology 60 (38.4%) followed by microbiological diagnosis 39 (25%). Table 4 shows the details of microbiological diagnosis in PTB and EPTB.

**Table 4:** Bacteriology results of PTB, EPTB and disseminated TB.

Type of TB	Culture		AFB smear and GXP results				
	Results	No. (%)	Sm+ve/GXP+ve n(%)	Sm-ve/GXP+ve n (%)	Sm+ve/ GXP-ve n (%)	Sm-ve/GXP-ve n (%)	NA n (%)
**PTB** **n = 96**	**Total**	**96 (100)**	**48(50)**	**22 (22.9)**	**5(5)**	**21(21.8)**	
	Positive	35(36.4)	28/35(80)	6/35(17)	–	1/35(2.8)	
	Negative	61(63.5)	20/61(32.7)	16/61(26)	5/61(5)	20/61(20.8)	
**EPTB** **n = 156**	**Total**	**39(25)**	**4/39(10)**	**28/39(77)**	**5/39(13.8)**	**2/39(5)**	
	Positive	9/39(23)	2/9(22)	5/9(55.5)	0	2/9(22)	
	Negative	30/39(76.9)	2/30(6.6)	23/30(76.6)	5/30(16.6)	0	
	Not done	117/156 (75)					117(75)
**Disseminated** **n = 7**		**3/7(42.8)**					
	Positive	2/3 (66.6)	2	–	–	–	
	Negative	1/3 (33)	–	1	–	–	
	Not done	4/7(57)					4(57)

## Discussion

This is the largest study on patients with TB and CKD from Pakistan. We found 60% of our patients presented with EPTB. A nationwide multi-center study from Pakistan reported the frequency of EPTB in general population as 29.2% [10]. Xiao et al compared clinical characteristics in CKD and non CKD patients and found EPTB incidence of 41-66.6% in pre hemodialysis or hemodialysis patients [1]. Studies from Nepal and Taiwan also noticed a higher proportion of EPTB (69.1% and 51.6% respectively) among CKD patients [11,12]. Similarly a small study from Pakistan on hemodialysis dependent patients reported EPTB as 79% of all TB cases [13]. Since EPTB is a re-activation of latent TB and patients with immune dysregulation are more at risk of reactivation, HIV or those on immunosuppressive medications have higher incidence of EPTB than in the general population [14]. Similarly advanced renal failure causes persistent inflammation leading to functional abnormalities of B and T cells. This may be the cause of increased risk of EPTB in this patient population [3]. An epidemiological data from US also found a significant association of renal failure with EPTB [15]. We observed higher proportion of EPTB in patients other than CKD 4 & 5 also. This may be due to the fact that majority of our patient population are either with renal failure, cancer or other chronic diseases, who may be partially immunocompromised, this may the reason to observe similar proportion of EPTB in this population.

In this study we found pleural, lymph nodes and urogenital system as a predominant presentation of EPTB. Our results are consistent with previous reports both from endemic and non-endemic areas in which pleural TB and TB lymphadenitis are major sites involved in CKD patients [11,16]. In contrast, genitourinary TB is one of the common presentation in current study due to the fact that SIUT is the largest urology and nephrology center of Pakistan where patients with genitourinary TB were referred. The diagnosis of EPTB is difficult because of the atypical presentation and pauci-bacillary disease [17]. In our study we were able to diagnose EPTB in 25% of patients through microbiology with 6% culture positive cases. A nationwide study from Pakistan reported only 8.8% of EPTB diagnosed on microbiology [10]. This may be because of the presence of an active department of Infectious Diseases in our center which raised the awareness of microbiological diagnosis of TB by practice and teaching. As a result primary physicians sent samples like urine, tissue or fluid for ZN stain, GXP and culture promptly and hence we were able to diagnose EPTB in one fourth of our patient population. Furthermore, in PTB we also observed a higher yield of positive GXP in our cohort, around 70% as compared to a yield of 40–50% reported in literature [1,12]. However, TB culture yield was 17% in our center as compared to 20–23% reported in the literature [18]. This may be because we have a higher number of extrapulmonary tuberculosis where we were unable to send samples for microbiological diagnosis.

We found INH resistance of 21% in culture positive cases, although only 6% of our patients had relapsed TB. A large retrospective laboratory based surveillance data of general population from Pakistan reported an INH resistance of 10–15% in 2019 [19]. Drug resistance data in renal failure patients is sparse. A retrospective study on dialysis and renal transplant recipients found INH resistance in 19% of patients [16]. Ostermann et al from UK reported INH resistance in 25% of culture positive TB cases among CKD patients [20]. This is an area of concern. We recommend to introduce GXP testing for detection both rifampicin and INH genes in order to diagnose INH resistance promptly and treatment tailored accordingly.

Despite high INH resistance, we were able to achieve successful treatment response in 81% of our patients and there was no treatment failure. Baghai et al from Iran also reported no treatment failure in CRF patients [21]. A study from China reported a 68% success in pre-hemodialysis patients [1].

The all-cause mortality was 15% in our cohort. Overall the mortality rate of CKD patients with TB is much higher than in general population. Qureshi et al reported a mortality rate of 7% in hemodialysis patients from Pakistan [13]. A study from Australia compared all-cause mortality among different stages of CRF, they found a significant increase in mortality among those with lower glomerular filtration rate [22]. A report from Nepal in 49 diagnosed TB patients with CKD found to have 28.6% mortality within 2 months [11].

Several risk factors have been identified for poor prognosis in patients with compromised renal functions such as age ≥ 40 years, hypoalbuminemia and CKD stage 4-5 [1]. In contrast we have found pulmonary TB as a significant risk factor associated with mortality. A study from Iran on general population did find smear positive TB as a significant risk factor for mortality [23].

When patients with CKD stage 4 & 5 were compared with other patients including patients with CKD stage 1-3. We found significantly higher mortality and a lesser treatment success rate in these patients. A study from China also reported similar findings with significant high mortality among pre-hemodialysis and hemodialysis patients [1]. Another study from Iran also reported a poor outcome in patients with renal failure [21].

Limitation of our study is that, it is a retrospective observational study due to which there may be some missing data. However, to the best of our knowledge this is the first study with a large data on TB in CKD from Pakistan.

In conclusion, EPTB is the most common presentation in patients with CKD stage 4 & 5. We were able to get the microbiological diagnosis in one fourth of the patients with EPTB. The treatment success is significantly low with high mortality and PTB is a significant risk factor for mortality. We found a very high INH resistance in our patient population which is an area of concern. We recommend to introduce GXP resistance gene testing for INH in order to diagnose INH resistance promptly.

## Supporting information

S1 Table Resistance among patients with culture Positive (n = 46). (DOCX)
