# Supplementary material for: Clinical presentation and outcome of tuberculosis in chronic kidney disease stage 4 & 5 from a high TB burden country
Source: PLoS One. 2025 Apr 2;20(4):e0320907. doi: 10.1371/journal.pone.0320907 (PMC11964231; doi:10.1371/journal.pone.0320907)
Supplement: S1 Table — (DOCX) [file pone.0320907.s001.docx]

| **Drug resistance** | 16 (34.7) |
| --- | --- |
| Mono resistant | 12 (26) |
| Poly resistant | 4 (8.6) |
| **Individual drug resistance**  Isoniazid  Rifampin  Pyrazinamide  Ethambutol | 10 (21.7)  1 (2.1)  4 (8.7)  5 (10.9) |

Table S1: Resistance among patients with culture Positive (n=46)
